# The validation of quality attributes in Primary Human Hepatocytes Standard

**DOI:** 10.1186/s13619-025-00258-6

**Published:** 2025-10-16

**Authors:** Zhaoliang Peng, Jiaying Wu, Xi Zhang, Xinyang Jia, Zhitao Wu, Hao Dai, Da Huang, Xin Cheng, Guoyu Pan, Ruimin Huang

**Affiliations:** 1 Shanghai Institute of Materia Medica, Chinese Academy of Sciences, 555 Zuchongzhi Road, Shanghai, 201203 China; 2 Center for Excellence in Molecular Cell Science, Chinese Academy of Sciences, 320 Yueyang Road, Shanghai, 200031 China; 3 School of Chinese Materia Medica, Nanjing University of Chinese Medicine, Nanjing, 210029 China; 4 EndoCell Therapeutics, Inc, Shanghai, China

**Keywords:** Primary human hepatocyte, Cell quality attribute, Hepatocyte marker, Single cell transcriptomic analysis, Drug metabolism function

## Abstract

Primary human hepatocytes (PHH) are used as the FDA-recognized "gold standard" for liver-related studies in vitro. The world’s first PHH group standard (T/CSCB 0008–2021, CSCB standard) was released by Chinese Society for Cell Biology in 2021. In order to justify this standard, six key quality attributes of ten different batches from commercial PHHs, including cell viability, cell morphology, cell markers, albumin secretion, drug metabolism function and bile secretion, were characterized using the designated test methods in the standard. The PHHs from various batches all exhibited typical hepatocytic morphology, high cell viability, and sufficient albumin secretion; whereas, tremendous variations in cell markers, drug metabolism functions, and bile secretion were unexpectedly detected across the board. Flow cytometric assessment of hepatocyte markers revealed the percentages of ALB^+^ or HNF4A^+^cells in six batches of PHHs, ranging from 49.4% to 98.9% and from 37.7% to 91.4%, respectively. Single cell transcriptomic analysis also revealed significant cell heterogeneity across the different batches, with the proportions of hepatocytes ranging from 69.2% to 98.9%. Considerable heterogeneity in drug metabolism functions across the batches were also found in substrate clearance rate (SCR) and metabolite formation rate (MFR) for six representative CYP450 enzymes, while the results didn’t influence current SCR attribute of CYP3A4. Metabolic capacity and purity are two independent attributes for PHH. The varied biliary excretion indexes around criteria (30%) indicated heterogeneity of PHH biliary excretion capacity. These results confirmed the robustness of most quality attributes in current CSCB standard, while highlighting the need to refine remaining parameters to enhance its practical applicability.

**Supplementary Information:**

The online version contains supplementary material available at 10.1186/s13619-025-00258-6.

## Background

The liver is a key organ that modulates various vital physiological processes, regulating plasma glucose and ammonia levels, maintaining lipid and cholesterol homeostasis, detoxifying drugs, synthesizing bile, storing and processing key nutrients, and supporting immune system (Driskill and Pan 2021; Trefts et al. 2017). The functions of liver can be compromised by a wide range of factors, such as drug-induced hepatotoxicity and infectious or metabolic diseases (Devarbhavi et al. 2023; Polidoro et al. 2021). The parenchymal cells, including hepatocytes and cholangiocytes, are the principal constituent cells and functional bearers of the liver. A variety of liver diseases are directly linked to the damages of liver parenchymal cells; thus, the appropriate cell models that enable faithful recapitulation of liver physiological complexity for deciphering disease-related mechanisms are crucial.

Currently, a variety of models involving cultured liver cells, including primary hepatocytes (PHHs), hepatocellular carcinoma cells and the pluripotent stem cell-derived hepatocytes, have been established. PHHs isolated directly from liver tissue are able to maintain, at certain levels, the major intrinsic liver functions, and therefore have served as the FDA-approved"gold standard"in vitro model for liver-related studies (Gomez-Lechon et al. 2014). HepaRG cells are terminally differentiated hepatocytes deriving from a human hepatic tumor cell line that retains a panel of major characteristics of PHHs (Aninat et al. 2006), but their application is undermined by the tumorigenic origin and the single genetic background that fails to represent the genetic diversity in human beings (Mann 2015). Human hepatocellular carcinoma cells, including HepG2 or Huh7, are frequently used as liver cancer models; while their utility on metabolism and detoxification evaluation is limited due to the low expression of drug-metabolizing enzymes (Guo et al. 2011). The pluripotent stem cell-derived hepatocytes (hPSC-Heps) are well-known for their unlimited source, and therefore representing a promising alternative to the conventional PHH or hepatocellular carcinoma cells-based in vitro liver models (Takayama et al. 2013); but their application is hampered by the fact that hPSC-Heps generated from the existing in vitro differentiation systems are frequently not developmentally and functionally mature (Takayama et al. 2012).

As PHHs have been commercialized globally and been widely used as an essential in vitro model for a variety of academic and industrial purposes, the Chinese Society for Cell Biology released the first PHH group standard (T/CSCB 0008–2021) worldwide (namely, CSCB standard) on 9 January 2021 (Peng et al. 2022). This standard delineates the technical requirements for PHHs, in particular, the primary quality attributes including cell morphology, cell viability, cell markers, albumin secretion, drug metabolism function, bile secretion, and microorganisms. However, this standard is empirically proposed based on the prior parameters presented by multiple academic and industrial groups, reflecting the unique perspectives or preferences of these two cohorts (Godoy et al. 2013). Despite the differential focuses, the people using PHHs all have concerns about the batch-to-batch variability of the available PHH products and the application range of current attributes. Therefore, it is essential to systematically validate these quality attributes across the PHH batches from different manufacturers, donors, storage durations and etc. Besides, in the CSCB standard, the potential correlations of the biomarkers and the correlation between the hepatocyte purity and the overall function have not been addressed. For example, the functional protein Albumin (ALB) (Quinlan et al. 2005; Aizarani et al. 2019) and the key hepatic transcription factor Hepatocyte Nuclear Factor 4 Alpha (HNF4A) (Yeh et al. 2019; Lemaigre FP 2009) are used as the major hepatocytic biomarkers in this standard, with the percentages (≥ 90%) of ALB or HNF4A positive cells being set as the purity criteria for PHHs. However, it has not been carefully investigated whether or not there is an expressional correlation between ALB and HNF4A and whether or not there is a correlation between the hepatocyte purity and the drug metabolism function. Finally, as only the intrinsic clearance rate (CL_int_) of testosterone, a substrate of CYP3A4, was defined as the representative drug metabolism ability of PHHs in CSCB standard, it remains unclear whether the functions of other key CYP450 enzymes, i.e. CYP1A2, CYP2B6, CYP2C9, CYP2C19, and CYP2D6 (Laine et al. 2009; Faucette et al. 2000), should be evaluated.

To address the aforementioned questions, in this study the key quality attributes from the CSCB standard were evaluated in 10 batches of commercial PHHs from various manufacturers, using the designated methods. Besides, single cell transcriptomic analyses (scRNA-seq) were performed to assess the cell composition, the hepatocyte subpopulations and the expression levels of key hepatic genes across the PHH batches. Particularly, the correlations between the mRNA levels of *CYP450* family members/protein presence levels of ALB and HNF4A/hepatocyte proportion/secreted ALB level and the drug metabolism functions were investigated.

## Results

PHHs generated by four manufacturers from 10 donors (7 males and 3 females; 0.5~51 years old) with different storage durations (2~10 years) (Table 1) were evaluated in this study. The safety of these batches was assessed based on their quality inspection reports provided by manufactures, which only included the inspection results of viruses rather than those of bacteria, fungi, mycoplasma, or treponema pallidum (Table S1). The six key quality attributes listed in the CSCB standard were then investigated.

**Table 1:** Basic information of PHHs in this study

Batch #	Manufacturer	Catalog#	Lot #	Storage duration (year)	Donor age (year)	Gender
PHH330	BD-Discovery Labware	454543	330	10	49	female
PHH393	Corning-Discovery Labware	454541	393B	8	29	female
PHH396		454541	396	8	51	male
PHH409		454543	409	6	51	male
PHH416		454543	416	6	43	male
PHH005	Novabiosis	/	SQY005	4	25	male
PHH910	LifeNet Health LifeSciences	/	LHum15910	4	44	male
PHH211		/	1922211-01	2	40	male
PHH025		/	2011025-01	2	3	female
PHH789		/	2016789-01	2	0.5	male

### Cell viability of PHHs

In all ten batches of PHHs, ≥ 85% cells were viable after resuscitation, which met the requirement (≥ 70%) for cell viability in the CSCB standard.

### Cell morphology of PHHs

PHHs exhibited a polygonal morphology with a diameter of 20 to 30 μm. The majority of the cells were mono-, bi- or multi-nucleated, and the tight junctions could be observed at the cell borders (Fig. S1A), which are consistent with the morphology described in the CSCB standard.

### Expression of hepatic biomarkers in PHHs

Since the expressions of ALB and HNF4A have been recognized as the hallmarks of hepatocytes, they were assessed in 6 batches of PHHs by flow cytometry (FACS) (Table S2). Tremendous variations in the expressions of ALB and HNF4A were observed. The percentages of ALB^+^ cells ranged from 49.4% (PHH211) to 98.9% (PHH789), with the proportions of HNF4A^+^ cells ranging from 37.7% (PHH211) to 91.4% (PHH409). Apparently, except two batches (PHH409 and PHH416), the majority of these values were both lower than those defined ones described in the CSCB standard as “the expression of ALB and HNF4A shall be ≥ 90% of the cell population”. In addition, good correlation was found between ALB and HNF4A positive percentage (r = 0.89).

**Fig. 1:**
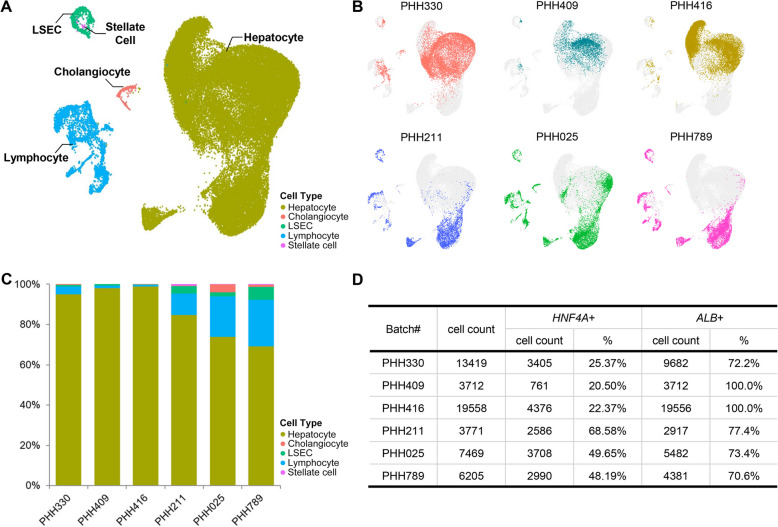
Single cell transcriptomic analysis on six PHHs, including PHH330, PHH409, PHH416, PHH211, PHH025, and PHH789. **A** UMAP clustering of scRNA-seq identified five cell types (hepatocyte, cholangiocyte, lymphocyte, LSEC, and stellate cell) in six PHHs. **B** Distribution of six PHHs in UMAP clustering. **C** The proportion of different cell types in six PHHs. **D** Cell counts and proportions of *HNF4A*^+^ (CPM > 0) or *ALB*^+^ (CPM > 200) cells in six batches of PHHs

scRNA-seq was employed to doublecheck the cell compositions and the expression of key hepatic genes in subpopulations of the 6 batches of PHHs that were assayed by FACS. The unsupervised clustering and differentially expressed genes (DEGs) analyses revealed major cell clusters that were defined as hepatocytes (the largest cluster), lymphocytes, liver sinusoidal endothelial cells (LSECs), cholangiocytes, and stellate cells, according to their characteristic gene expression patterns (Figs. 1A, S2A). Notably, three out six batches of PHHs (PHH211, PHH025, and PHH789) had more than 10% non-hepatocytes, the majority of which were of lymphoid lineages (Figs. 1B, C and S2B). In particular, there were more than 23% lymphocytes and 6% LSECs in PHH789.

**Fig. 2:**
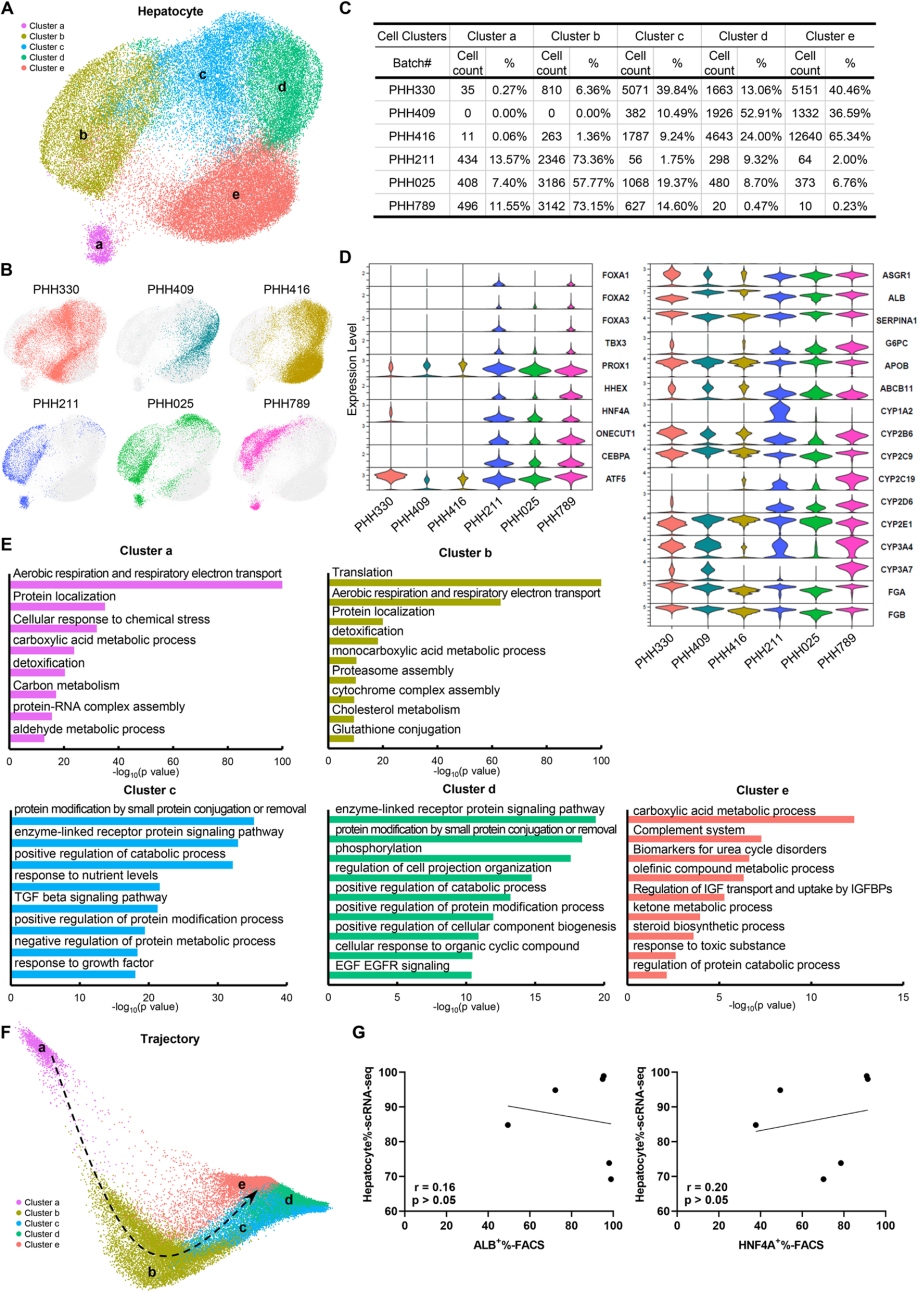
Delineation of hepatocyte subsets across the six PHHs. **A** UMAP clustering on the hepatocyte population identified in PHH samples indicating five cell clusters (Clusters a to e). **B** Distribution of hepatocytes from six PHHs in UMAP clustering. **C** Cell counts and proportions of different clusters in hepatocyte population from six PHHs. **D** Violin plots showing the expression levels of hepatic key transcriptional factors and functional genes in hepatocyte population from six PHHs based on scRNA-seq. **E** Selected GO terms enriched in each cluster based on differentially expressed genes. **F** Diffusion map showing the pseudo-time trajectory on hepatocyte population from six PHHs. **G** Correlation between hepatocyte% by scRNA-seq and ALB^+^% (left panel)/HNF4A^+^% (right panel) by FACS in six PHHs

It has been well-established that hepatocytes are heterogenous not only during embryonic and fetal development but also among adult individuals, and zonational pattern of mature hepatocytes have been recently studied in depth by lineage tracing and single cell transcriptomic analysis (Aizarani et al. 2019; Wang et al. 2020; Ben-Moshe and Itzkovitz. 2019; MacParland et al. 2018). Therefore, it is important to delineate hepatocytic subsets across various batches of PHHs, which may help to reveal the underlying batch-to-batch variations. scRNA-seq revealed 5 distinct subsets of hepatocytes as well as the apparently differential distribution of 6 batches of PHHs (Figs. 2A-C) which represented the heterogeneity of PHH batches in a deeper resolution. Each of these subsets had distinct expression pattern of key hepatic transcription factors, surface proteins, metabolic enzymes and other hepatocyte-specific proteins (Figs. 2D, S3), indicating differential roles or development stages for these subpopulations of hepatocytes. For example, while both cluster a and cluster b were mainly responsible for aerobic respiration and respiratory electron transport and detoxification, cluster a appeared more capable of carbon metabolism rather than cholesterol metabolism, the functional assignment to cluster b. Similarly, while cluster c and cluster d were both responsible for protein modification, cluster d appeared to be particularly competent for phosphorylation and sensitive to organic cyclic compounds and EGF, but cluster c appeared to be responsive for TGFβ and growth factors. Interestingly, cluster e was enriched for the components of the complement system (Fig. 2E). While PHH211, PHH025, and PHH789 were similar in subset distribution, the other three batches (PHH330, PHH409 and PHH416) were significantly different from one another, representing the variegated function capacities/preferences across the batches. Finally, the putative development trajectory inferred from the scRNA-seq data (Fig. 2F) indicated that the two batches of PHHs derived from two youngest donors (PHH789 0.5-year and PHH025 3-year) were closely linked to each other (Figs. 2B, D), revealing similar developmental stages and function maturations. In order to explore the correlations between hepatocyte proportion and the presence levels of hepatic biomarkers (ALB and HNF4A), analyses were conducted based on hepatocyte percentage (hepatocyte%-scRNA-seq) decided by the hepatic marker signature in scRNA-seq and ALB^+^%/HNF4A^+^% by FACS. Very weak positive correlations without statistical significance were observed (r = 0.16 for ALB and r = 0.20 for HNF4A, p > 0.05; Fig. 2G). Taken together, these data revealed significant heterogeneities among PHH batches.

### Secretion levels of ALB in PHHs

**Fig. 3:**
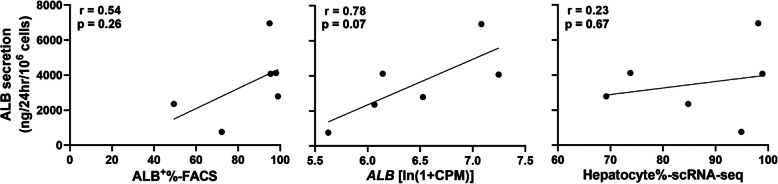
Correlations between normalized secreted albumin level and the different hepatocyte markers, including ALB^+^% (by FACS), ALB mRNA level (by scRNA-seq), and hepatocyte% (by scRNA-seq), in six PHHs

Secreted ALB was detected in 6 batches of PHHs by enzyme-linked immunosorbent assay (ELISA) as the CSCB standard suggested, ranging from 762.7 ± 174.1 to 6957.7 ± 2440.5 (ng/24 h/10^6^ cells) (Table S3), which met the requirement of the CSCB standard (≥ 800 ng/24 h/10^6^ cells), expect PHH330. The normalized secreted albumin level was compared with the corresponding hepatocyte markers, including ALB^+^% values from FACS, ALB mRNA level and hepatocyte% from scRNA-seq. Strong positive correlation could only be found between ALB excretion level and its mRNA level (r = 0.78) but without statistical significance (p = 0.07) (Fig. 3), indicating the relatively weak correlations among these features.

### CYP450 metabolic capacities in PHHs

In the CSCB standard, three drug metabolic enzymes (CYP1A2, CYP2B6, and CYP3A4), each belonging to three major subfamilies of CYP450, were recommended to assess the metabolic capacities, with the corresponding substrate/metabolite pairs being phenacetin (PHE)/4-acetaminophenol (APAP) for CYP1A2, bupropion (BUP)/4-hydroxybupropion (4-OH-BUP) for CYP2B6, and testosterone (TE)/6β-hydroxytestosterone (6β-OH-TE) for CYP3A4, respectively. However, only the CL_int_ rates of substrates were required in CSCB standard. In order to “meta-analyze” the drug metabolic function of PHH, three additional CYPs (CYP2C9 by diclofenac (DIC)/4’-hydroxydiclofenac (4-OH-DIC), CYP2C19 by mephenytoin (MEP)/4-hydroxymephenytoin (4-OH-MEP), and CYP2D6 by dextromethorphan (DXM)/dextrorphan (DXO) were investigated, and both substrate clearance rate (SCR) and metabolite formation rate (MFR) for each enzyme were assessed.

For the six selected CYP450 enzymes, SFR (Table S4) and MFR (Table S5) were examined using the detection parameters by LC-MS in 6 batches of PHHs (Table S6). For CYP3A4, the TE clearances were well above CSCB standard (≥ 100 μL/hr/10^6^ cells) in all 6 batches, so were those of BUP, a substrate for CYP2B6’s. For CYP2D6 and CYP2C9, most batches showed substrate clearances either higher than or slightly lower than 100 μL/hr/10^6^ cells. In contrast, the clearance rates of PHE (a substrate for CYP1A2) and MEP (a substrate for CYP2C19) varied a lot across the batches, ranging from undetectable to more than 200 μL/hr/10^6^ cells (Table S4). From the perspective of MFR, the generation levels of APAP (by CYP1A2), 4-OH-BUP (by CYP2B6), 4-OH-DIC (by CYP2C9), DXO (by CYP2D6) and 6β-OH-TE (by CYP3A4) were relatively high in all batches except PHH025; whereas the generation level of 4-OH-MEP (by CYP2C19) was relatively low or even undetectable in all batches except PHH789 (Table S5).

**Fig. 4:**
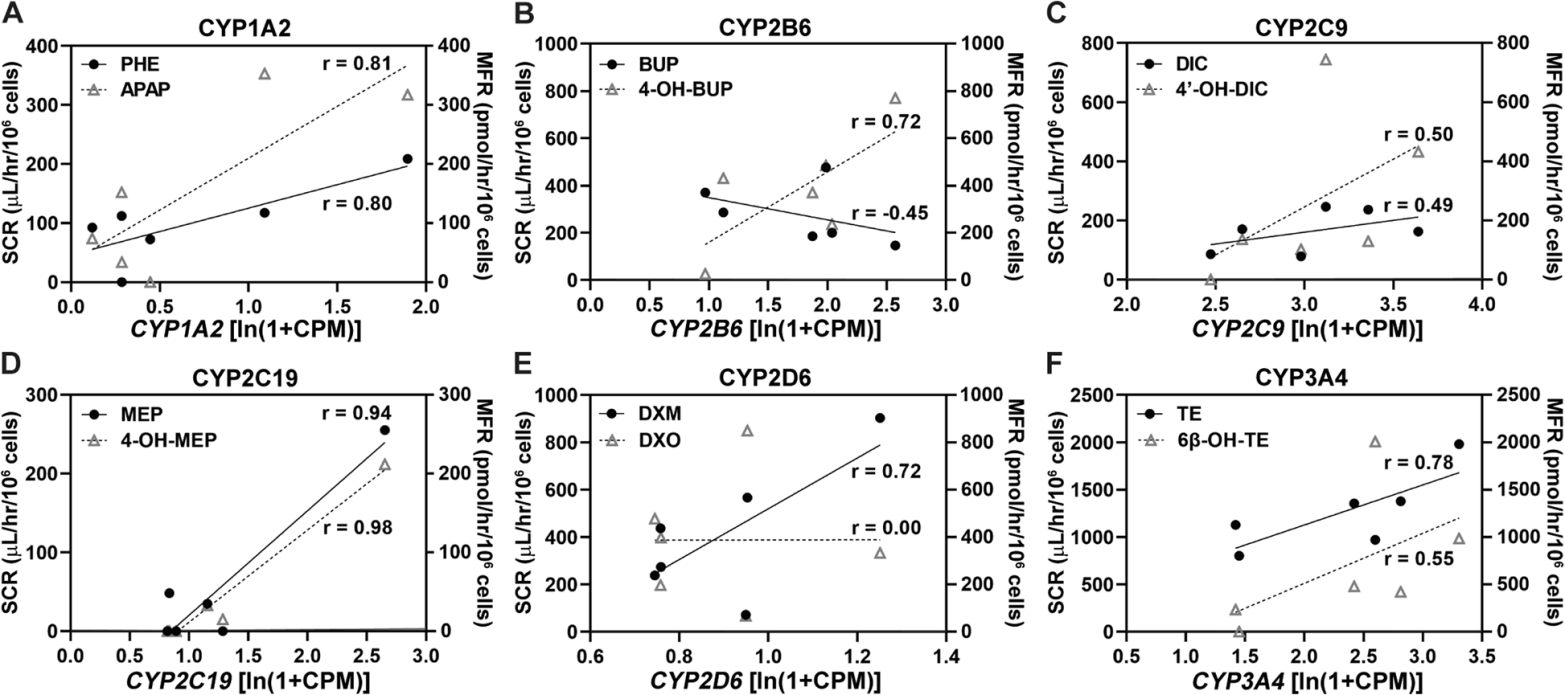
Correlations between CYP450 enzyme expression level by scRNA-seq and the corresponding substrate clearance rates (SCR; solid dot or solid line) or metabolite formation rates (MFR; hollow triangle or dotted line) in six PHHs. r, Pearson correlation coefficient

To determine whether or not the aforementioned CYP450 enzyme activities were influenced by their expression levels, the correlations between the mRNA levels of *CYP450* enzymes and their SCR or MFR levels were calculated using Pearson correlation analysis (Fig. 4). The association strength was defined as: low (0.1 < r < 0.3 or -0.3 < r < -0.1), medium (0.3 ≤ r ≤ 0.5 or -0.5 ≤ r ≤ -0.3), and high (0.5 < r < 1.0 or -1.0 < r < -0.5). Both SCR and MFR for CYP1A2 showed high correlations with *CYP1A2* mRNA levels (Fig. 4A), and the same for CYP3A4 (Fig. 4F). Only SCR for CYP2D6 (Fig. 4E) and only MFR for CYP2B6 (Fig. 4B) had high-strength associations with their mRNA levels. For CYP2C19, since the substrate and metabolite were undetectable in three batches, the correlation was not established even though r > 0.5 (Fig. 4D).

The CYP450 enzyme activities were further analyzed for their potential correlations with the percentages of ALB^+^ (Fig. S4) or HNF4A^+^ cells (Fig. S5) determined by FACS, the proportion of hepatocytes (Fig. S6) estimated by scRNA-seq, and the ALB secretion (Fig. S7) by ELISA, respectively. No significant correlations were detected in any of these parameter pairs. These data indicated that enzyme activities (both SCR and MFR) of CYP1A2 and CYP3A4 had high correlations with their mRNA levels.

### Bile secretion index (BEI) in PHHs

In the CSCB standard, BEI, deuterium-labeled sodium taurocholate (d8-TCA) as the substrate, is used as the attribute for bile secretion evaluation. Using the designated method in CSCB standard, BEI was assessed in 5 batches of PHHs (PHH393, PHH396, PHH005, PHH910, and PHH416) cultured in a sandwich-system for 5 days (Figs. S1B, C). Comparing with recommended BEI value (≥ 30%) in the CSCB standard, only 2 out of 5 batches of PHHs showed BEI values significantly than 30%, while the rest were very close to 30% (Table S7). These findings suggested that PHHs in sandwich culture could sustain polarity, generate networks of bile canaliculi, and exhibit varied biliary excretion capacities.

## Discussion

The CSCB standard (T/CSCB 0008–2021) is the first globally available standard for PHHs (Peng et al. 2022). In order to ensure the quality control of PHHs products, the CSCB standard outlines technical requirements, test methods, test regulations, instructions for use, labelling requirements, packaging requirements, storage requirements and transportation requirements for PHHs. Although this standard is established on the publications and expertise of multiple academic and industrial groups, it has not been validated in a comprehensive manner yet. In this study, ten batches of commercially available PHHs from various vendors were utilized to evaluate the six primary quality attributes (cell viability, cell morphology, cell markers, ALB secretion, drug metabolism and bile secretion capacities) listed in the CSCB standard. Our results have confirmed the robustness of most quality attributes in current version, and also demonstrated the necessity for the refinement of the several key parameters, which is important for the CSCB standard to conform to the reality of the commercially available PHHs.

Our results demonstrated that the tested PHH batches exhibited normal hepatocyte morphology, high cell viability and sufficient albumin secretion, which satisfied the CSCB standard’s requirements. However, tremendous variations across the batches were observed in the proportions of ALB^+^ or HNF4A^+^ cells, as well as the drug metabolic and bile secretion capacities, which indicates that considerable heterogeneities existed among different batches of PHHs, possibly due to donor diversity, or differential isolation protocols, transportation and storage conditions/durations.

The purity of hepatocytes is an essential parameter for the quality control of PHHs, as higher purity is expected to be associated with more potent drug metabolic and bile secretion functions. It was once believed that PHH products should have relatively high purity although it has not been validated comprehensively. To our surprise, the hepatocyte purities varied greatly across the batches when assayed by either FACS (Table S2) or scRNA-seq (Figs. 1C, S2B), and in some cases were far below “90%” as stipulated in the CSCB standard. The possible reasons for this discrepancy may come from various factors, including hepatocyte heterogeneity as well as the differences of detection objects. 1) Hepatocyte subpopulations in diverse functional states are characterized by intrinsic variations in gene expression. This biological heterogeneity could introduce the weak correlation between ALB^+^%/HNF4A^+^% and hepatocyte% potentially due to donor-derived individual differences. 2) ALB^+^%/HNF4A^+^% was determined by flow cytometry for ALB/HNF4A protein presence; whereas hepatocyte% was predicted by clustering and threshold-based partitioning grounded in transcriptomic features. Consequently, discrepancies between the outcomes of these two approaches are not surprising, in that it is unnecessary that protein presence correlates linearly with mRNA expression.

scRNA-seq is a very powerful tool to dissect the cell subpopulations in a cell mixture, such as PHHs. We have found 5 cell populations in each batch of PHH, and identified 5 major hepatocyte subpopulations that differentially existed in various batches of PHHs, representing the donor diversity in particular the age/developmental differences. The five hepatocyte subsets differed in either developmental statuses or functional preferences, and might account for the functional variations observed across the tested batches. Notably, among the three batches (PHH211, PHH025, and PHH789) that were similar in subset distribution, PHH789 from the youngest donor was, as expected, particularly enriched for *CYP3A7*, a CYP450 enzyme that is mainly expressed by fetal hepatocytes (Fig. 1D) (Shum and Isoherranen 2021). In addition, these three batches expressed *CYP2C19*, while the rest three batches (PHH330, PHH409 and PHH416) did not (Fig. 1D), which was consistent with the high SCR for MEP (a substrate of CYP2C19) in PHH211, PHH025, and PHH789 (Table S4). An interesting finding was that the expression levels of the key hepatic transcription factors (*FOXA1/2/3*, *TBX3*, *PROX1*, *ONECUT1* and *HNF4A*) were significantly higher in PHH211, PHH025 and PHH789 than those of the rest three batches, suggesting regulatory roles of these transcription factors in hepatocytic function subsets.

Drug metabolism function of PHHs is often utilized for in vitro drug screening. In the CSCB standard, CL_int_ of a specific substrate is often applied to evaluate the activity of the corresponding CYP450 enzyme. In this study, activities of six CYP450 enzymes were assessed by both SCR and MFR. Significant heterogeneity on drug metabolism functions were observed. It was found SCR and MFR had strong correlations with the mRNA levels of the corresponding CYP450 for CYP1A2 (Fig. 4), while for CYP3A4, high correlation is for SCR only, not MFR. The results suggested that SCR of CYP3A4, rather than MFR, can serve as a reliable criterion for current CSCB standard.

Another intriguing finding was the inconsistency of metabolic capacity with other major attributes of PHHs. In this study, no correlation was found between the CYP450 enzyme activities and the hepatocyte purity, or ALB secretion, or *ALB*/*HNF4A* mRNA levels (Figs. S4-7). One potential explanation was there may exist considerable redundancy in the metabolic capacity of hepatocytes, even cell purities/protein expression levels varied greatly. In other words, CYP450 enzymes in hepatocytes were enough to metabolize all the substrates supplied in a short time (To calculate SCR, in most cases, the substrate concentration should be far below the enzyme’s saturation concentration (i.e., [S] < < Km)). The current metabolic capacity of the system is adequate to assess SCR, indicating that cell purity or other factors did not exert a significant effect on metabolic capacity. Therefore, even when hepatocytes purity is relatively low, or albumin excretion capacity is not high, the situation won’t influence the overall metabolic capacity of hepatocytes tested. It is unnecessary to establish a linear relationship between cell purity and metabolic capacity.

Biliary excretion is one of the most important physiological processes for the detoxification or elimination of drugs and metabolites. Evaluating the hepatobiliary transport of drugs is essential in drug discovery stage (Ghibellini et al. 2006). In this context, the sandwich-cultured hepatocytes are recognized as a conventional tool to evaluate the biliary excretion of drugs (Swift et al. 2010). The sandwich culture involves the encapsulation of primary hepatocytes between two layers of gel-collagen matrix, offering notable advantages over traditional 2D monolayer cultures. These advantages include enhanced hepatocyte morphology and viability, prolonged maintenance of functional activities, restoration of cell polarity that facilitates the correct localization of basolateral and tubular transporters, and the promotion of a functional bile duct network (Godoy et al. 2013). The hepatocytes cultured in sandwich system are capable of forming functional bile duct network which possess the ability to secrete bile (Pan et al. 2012). The BEI assay is generally used to evaluate whether the tested drug is a substrate of efflux transporters (Qiao et al. 2021). The tested compound would be considered to have high biliary excretion risk if its BEI in PHH is greater than 10% (Pan et al. 2012). For compound with high biliary excretion capacity, their BEI values could be much higher. For example, in multiple reports, the BEI values of TCA could be up to 50–70% (Wu et al. 2014; Ni et al. 2016; Zhou et al. 2017). In the CSCB standard, the BEI criterion for d8-TCA was set as greater than 30%, while, however, in this study, three out of five PHH batches displayed BEI values that were slightly lower than 30% (Table S7) which might be a result from multiple factors that can compromise PHHs biliary excretion (Xiang et al. 2019; Chen et al. 2021), including, but not limited to, extended storage duration (Table 1).

## Conclusions

In this study, a comprehensive investigation of established human hepatocyte quality attributes with multiple samples from real world was conducted. This research provided a novel perspective on the application of hepatocytes. The results confirmed the robustness of most quality attributes. However, unexpected characteristics have been identified, indicating some attributes need to be revised/refined or should be used in different circumstances:

1. Hepatocytes purity varied widely than expected. High heterogenicity of PHH makes it necessary to lower purity criteria from 90% to 70%.
2. A strong correlation between ALB^+^% and HNF4A^+^% was established in FACS assay, indicating both ALB and HNF4A could be used to assess hepatocytes purity.
3. scRNA-seq is a powerful tool to assess major attributes of PHHs.
4. SCR is still the best assay to assess hepatocyte metabolic capacity, especially for CYP3A4. CYP450 enzyme activity serves as an indicator, independent of purity and albumin expression/excretion, to evaluate hepatocyte quality.
5. Being a good indicator for biliary excretion capacity, BEI can however be affected by multiple factors, including storage conditions.

## Methods

### Chemicals and Reagents

APAP (MedChemExpress, HY-66005R), BUP (MedChemExpress, HY-B0403), 4-OH-BUP (MedChemExpress, HY-100637), DXM (Sigma-Aldrich, Y0002402), DXO (Absin, abs44112232), DIC (Sigma-Aldrich, SML3086), 4-OH-DIC (Sigma-Aldrich, 32412), MEP (MedChemExpress, HY-B1184), 4-OH-MEP (Sigma-Aldrich, H146), PHE (Sigma-Aldrich, 77440), TE (Sigma-Aldrich, T5411), 6β-OH-TE (Sigma-Aldrich, H-059), and d8-TCA (Toronto Research Chemicals, ABC-RC6107) were purchased from the indicated suppliers.

### PHH culture

Multiple batches of PHHs from 10 donors were obtained from BD Bioscience-Discovery Labware, Corning-Discovery Labware, Novabiosis, and LifeNet Health LifeSciences, whose information was summarized in Tables 1 & S1. Cryopreserved PHHs were resuscitated according to the manufacturers’ instruction. Briefly, PHHs were thawed at 37 °C as short as possible. After adding 5 mL InVitroGRO Thawing Medium (Rild-Biotech, S03317) with 10% fetal bovine serum (Gibco, A5669701), cells were centrifuged with 100 × g at room temperature for 5 min. Then cells were seeded into 12-well plates pre-coated with collagen I (1:1000 diluted by sterile ultrapure water; Corning, 354236) at a density of 1 × 10^6^ cells/well, and maintained in InVitroGRO Incubation Medium (Rild-Biotech, Z99009) at 37 °C with 5% CO_2_ for further evaluations on drug-metabolizing function.

### Cell viability

The viability of PHHs was determined by trypan blue staining. Equal volume of 0.4% trypan blue solution (Meilune, MA0130) and cell suspension were mixed and incubate 3 min at room temperature. 10 µL mixture was applied into a cell counting chamber slide (Invitrogen, C10283) and the percentage of viable cells was measured and calculated by the Countess Automated Cell Counter (Invitrogen, Countess 3).

### FACS

To assess ALB and HNF4A expression, 1-3 × 10^5^ single cells were subject to flow cytometry (BD Biosciences, FACSCelesta). Briefly, PHHs were fixed with 4% paraformaldehyde (Beyotime, P0099) and permeabilized with Immunostaining Permeabilization Buffer with Triton X-100 (Beyotime, P0096) for 15 min. Then cells were incubated with primary antibody at 4 °C for 30 min, followed by incubation with the corresponding secondary antibodies at 4 °C for 30 min. The antibodies were: anti-human albumin polyclonal antibody (1:200; Bethyl Laboratories, Cat# A80-129A, RRID: AB_2891968), anti-HNF4α (C11F12) rabbit mAb (1:100; Cell Signaling Technology, Cat# 3113S, RRID: AB_2295208), donkey anti-goat IgG (H + L) cross-adsorbed secondary antibody, Alexa Fluor 647 (1:400; Thermo Fisher Scientific, Cat# A-21447, RRID: AB_141844), and Alexa Fluor 488 AffiniPure donkey anti-rabbit IgG (H + L) (1:400; ImmunoResearch Laboratories, Cat# 711-545-152, RRID: AB_2313584).

### scRNA-seq

PHHs were loaded on a Chromium Single Cell Controller (10 × Genomics) to generate single-cell gel beads in emulsion (GEMs) using Single Cell 3 Library and Gel Bead Kit V2 (10 × Genomics, 120237) and Chromium Single Cell A Chip Kit (10 × Genomics, 120236) according to the manufacturer’s protocol. Then sequencing was performed on an Illumina Novaseq6000 with a sequencing depth (≥ 100,000 reads per cell) and pair end 150 bp (PE150). The Seurat package was finally used for data processing and analysis. The detailed experimental procedures and data processing methodologies were presented as below.

#### Cell capture and cDNA synthesis

Cell suspensions (300~600 living cells per microliter determined by Countstar) were loaded on a Chromium Single Cell Controller (10 × Genomics) to generate single-cell GEMs. Briefly, single cells were suspended in 0.04% BSA-PBS and ~ 8,700 cells were added to each channel with a targeted cell recovery estimate of 5,000 cells. Captured cells were lysed and the released RNA was barcoded through reverse transcription in individual GEMs. GEMs were reverse transcribed in a C1000 Touch Thermal Cycler (Bio-Rad) programed at 53 °C for 45 min, 85 °C for 5 min, and held at 4 °C. After reverse transcription, single-cell droplets were broken and the single-strand cDNA was isolated and cleaned with Cleanup Mix containing DynaBeads (Thermo Fisher Scientific). cDNA was generated and amplified, and quality was assessed using the Agilent 4200 TapeStation system.

#### scRNA-Seq library preparation

scRNA-seq libraries were prepared using Single Cell 3 Library Gel Bead Kit V2 following the manufacturer’s instruction. Sequencing was performed on an Illumina Novaseq6000.

#### Single-cell raw data processing

Illumina basecall files (*.bcl) were converted to fastqs using the Cell Ranger pipeline with recommended parameters. FASTQ files were aligned to GRCh38 human reference genome to generate the gene-cell unique molecular identifier (UMI) matrix. This output matrix was then imported into the Seurat (version 4.2.0) R toolkit (Hao et al. 2021) for quality control and downstream analysis. For each cell, we quantified the number of genes, UMIs and proportions of gene expression, and kept high quality cells with the following thresholds: 1) number of genes > 500, 2) UMIs > 600, and 3) percentage of mitochondrial genes < 20%.

#### Batch effect correction and normalization

Cell-specific network construction (CSN), a clustering method based on single-cell networks, was used to transform the initial gene expression matrix to network degree matrix (Dai et al. 2019), which could generate better clustering and dimensionality-reduction performance based on dynamic theory. Then, we performed batch correction across 6 samples (PHH330, PHH409, PHH416, PHH211, PHH025, and PHH789) by fastMNN method (Lun 2019; Haghverdi et al. 2018). This method performed a multi-sample principal component analysis and subsequent calculations in the principal component space instead of the original gene space, which optimized the computation speed and reduced the noise in neighbor detection (Tran et al. 2020). We applied the R package batchelor (version 1.12.3) to perform fastMNN, with the number of principal components (PCs) as 50 and the number of nearest neighbors in the same batch knn as 20. The output was 50 batch-corrected PCs and would be used as the input for further analyses.

#### Unsupervised clustering and cell type identification

An unsupervised graph-based clustering algorithm implemented in Seurat was applied to cluster all single cells. Function FindNeighbors and FindClusters were used to cluster all single cells based on the 50 batch-corrected PCs with a resolution of 0.5. Ultimately, based on their respective marker genes, the cells were classified into five distinct clusters, corresponding to five cell types: hepatocytes, cholangiocytes, LSECs, stellate cells, and lymphocytes.

#### Dimensionality-reduction and visualization

For dimension-reduction, function RunUMAP was used to perform uniform manifold approximation and projection (UMAP) analysis (Lim and Qiu 2023) on the 50 batch-corrected PCs and got 2D visualization graphics. Function DimPlot, FeaturePlot and VlnPlot were used to produce 2D UMAP scatter plots, gene expression scatter plots and gene expression violin plots, respectively.

#### Identification of DEGs

DEGs were identified based on Wilcoxon rank-sum test in function FindMarkers and FindAllMarkers from the Seurat package. We selected the genes with p < 0.01, absolute log_2_[fold change (after Laplace transformation)] > 0.5, and minimum fraction > 0.1 as DEGs.

#### Marker genes for cell population definition

Hepatocyte: *HNF4A, HNF6, CEBPA, ATF5, AAT, ALB, CYP3A4, CYP3A7, CYP1A2, CYP2B6, CYP2C9, CYP2E1, APOB, ASGR1, G6PC*; Cholangiocyte: *EPCAM, KRT18, KRT7, KRT19, SOX4, SOX9, CFTR, AQP1, MMP7*; LSEC: *CLEC14A, CCL14, MGP, SPARCL1, FCN2, CLEC1B, LYVE1*; Stellate cell: *ACTA2, COL1A1, TAGLN, RBP1*; and Lymphocyte: *CD45*.

### ELISA for ALB protein

Under regular 2D culture for 24 h, ALB protein level in the culture supernatants was determined by Human Albumin ELISA Kit (Bethyl Laboratories, E88-129) following the manufacturer’s instruction. The absorbance at 450 nm was measured by a microplate reader (Epoch 2, BioTek) and normalized by the numbers of PHHs. Mean ALB secretion of PHHs was calculated as follows: ALB secretion (ng/24 h/10^6^ cells) = A × V/E, where A was the sample ALB concentration determined according to the standard curve, V was the supernatant volume, and E was the cell number.

### LC-MS for drug-metabolizing function

Sample processing and analysis by LC-MS (Shimadzu, LCMS-8030) were referred to our previous publication (Peng et al. 2022). The CYP450 enzymes and their corresponding substrates/metabolites for drug-metabolizing function, along with the detection parameters in LC-MS, were summarized in Table S6. SCR was calculated as follows: SCR (μL/hr/10^6^ cells) = (0.693/T_1/2_) × (V/M), T_1/2_ (hr) = 0.693/K_1_. MFR was calculated as follows: MFR (pmol/hr/10^6^ cells) = (0.693/T_1/2_) × (V/M), T_1/2_ (hr) = 0.693/K_2_. The linear regression analysis of natural logarithm and time of the amount of substrate elimination or product generation yielded the regression equation Y = aX + b. The substrate elimination rate constant (K_1_) was equal to -a, while the product generation rate constant (K_2_) was equal to a. V/M was equal to the number of hepatocytes per incubation volume (10^6^ cells/mL).

### BEI assay

PHHs in sandwich culture were used for bile secretion index assay. 2.5 × 10^5^/well PHHs in OptiCulture hepatocyte medium (XenoTech, K8300) were seeded in the collagen I-coated 24-well plates and incubated at 37℃ with 5% CO_2_ overnight. Then, the medium was replaced with OptiCulture hepatocyte medium with 2% Matrigel (Corning, 356234) and changed daily for five days. Once bile duct-like structures were observed, PHHs were rinsed three times and pre-incubated with warm Hank’s balanced salt solution (HBSS) with/without Ca^2+^ (Gibco, 14025092/14175095) at 37 °C for 15 min, respectively. The supernatant was then removed, 5 μM d8-TCA in HBSS with Ca^2+^ was added for 15 min-incubation at 37 °C. The concentrations of d8-TCA in PHHs under the incubation condition with/without Ca^2+^ were assessed by LC-MS (Table S6). The BEI was calculated as follows: BEI (%) = (A_Ca_^2+^-A_Ca_^2+^_Free_)/A_Ca_^2+^ × 100%, where A_Ca_^2+^ was substrate concentration in PHHs under the incubation condition of calcium-containing HBSS, A_Ca_^2+^_Free_ was substrate concentration in PHHs under the incubation condition of calcium-free HBSS.

## Supplementary Information

Supplementary Material 1: Tables S1~S7, Figures S1~S7.

## Data Availability

The raw single cell RNA-seq data were deposited to the GEO database with the reference number GSE 289636 (https://www.ncbi.nlm.nih.gov/geo/query/acc.cgi?acc=GSE289636). All other data of this study are available from the corresponding authors upon reasonable request.

## Abbreviations

PHHs: Primary human hepatocytes
PSC-Heps: Pluripotent stem cell-derived hepatocytes
CSCB: Chinese Society for Cell Biology
ALB: Albumin
HNF4A: Hepatocyte Nuclear Factor 4 Alpha
CL_int_: Intrinsic clearance rate
scRNA-seq: Single cell transcriptomic analyses
FACS: Flow cytometry
DEGs: Differentially expressed genes
LSECs: Liver sinusoidal endothelial cells
ELISA: Enzyme-linked immunosorbent assay
PHE: Phenacetin
APAP: 4-Acetaminophenol
BUP: Bupropion
4-OH-BUP: 4-Hydroxybupropion
TE: Testosterone
6β-OH-TE: 6β-Hydroxytestosterone
DIC: Diclofenac
4-OH-DIC: 4’-Hydroxydiclofenac
MEP: Mephenytoin
4-OH-MEP: 4-Hydroxymephenytoin
DXM: Dextromethorphan
DXO: Dextrorphan
SCR: Substrate clearance rate
MFR: Metabolite formation rate
LC-MS: Liquid chromatography mass spectrometry
BEI: Bile secretion index
d8-TCA: Deuterium-labeled sodium taurocholate
GEMs: Gel beads in emulsion
PE150: Pair end 150 bp
UMI: Unique molecular identifier
PCs: Principal components
UMAP: Uniform manifold approximation and projection
HBSS: Hank’s balanced salt solution
