# Supplementary material for: The validation of quality attributes in Primary Human Hepatocytes Standard
Source: Cell Regen. 2025 Oct 16;14:41. doi: 10.1186/s13619-025-00258-6 (PMC12532551; doi:10.1186/s13619-025-00258-6)
Supplement: Supplementary file 1 — Supplementary Material 1: Tables S1~S7, Figures S1~S7. [file 13619_2025_258_MOESM1_ESM.docx]

**Supporting Information**

**The Validation of Quality Attributes in Primary Human Hepatocytes Standard**

Zhaoliang Peng^1†^, Jiaying Wu^2†$^, Xi Zhang^3^, Xinyang Jia^3^, Zhitao Wu^1,3^, Hao Dai^2^, Da Huang^2$^, Xin Cheng^2*^, Guoyu Pan^1,3*^, Ruimin Huang^1,3*^

^†^These authors contributed equally to this work.

^*^Correspondence

Xin Cheng, Center for Excellence in Molecular Cell Science, Chinese Academy of Sciences, 320 Yueyang Road, Shanghai 200031, China. Email: xcheng@sibcb.ac.cn

Guoyu Pan, Shanghai Institute of Materia Medica, Chinese Academy of Sciences, 555 Zuchongzhi Road, Shanghai 201203, China. Email: gypan@simm.ac.cn

Ruimin Huang, Shanghai Institute of Materia Medica, Chinese Academy of Sciences, 555 Zuchongzhi Road, Shanghai 201203, China. Email: [rmhuang@simm.ac.cn](mailto:rmhuang@simm.ac.cn)

**Table S1. The microorganisms report of PHHs from the manufacturers**


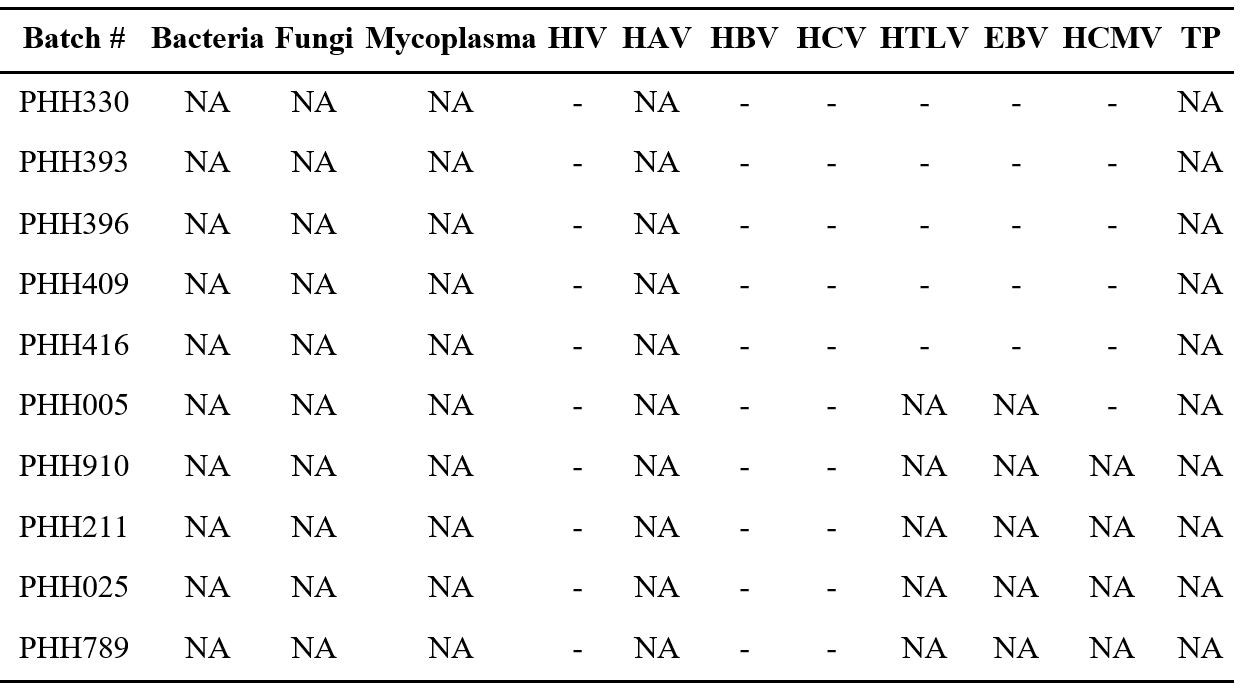


HIV: Human Immunodeficiency Virus;

HAV: Hepatitis A Virus;

HBV: Hepatitis B Virus;

HCV: Hepatitis C Virus;

HTLV: Human T-lymphotropic Virus;

EBV: Epstein-Barr Virus;

HCMV: Human Cytomegalovirus;

TP: Treponema Pallidum;

-: not detected;

NA: not applicable.

**Table S2. Percentage of ALB^+^ or HNF4A^+^ cells in PHHs by flow cytometry**

|  | **PHH330** | **PHH409** | **PHH416** | **PHH211** | **PHH025** | **PHH789** |
| --- | --- | --- | --- | --- | --- | --- |
| **ALB^+^ (%)** | 72.2 | 94.9 | 95.4 | 49.4 | 98.0 | 98.9 |
| **HNF4A^+^ (%)** | 49.4 | 91.4 | 90.9 | 37.7 | 78.5 | 70.2 |

**Table S3. Normalized secreted ALB by ELISA (ng/24 hr/10^6^ cells)**

|  | **PHH330** | **PHH409** | **PHH416** | **PHH211** | **PHH025** | **PHH789** |
| --- | --- | --- | --- | --- | --- | --- |
| **Mean ± SD** | 762.7 ± 174.1 | 6957.7 ± 2440.5 | 4076.1 ± 422.5 | 2358.7 ± 742.6 | 4122.0 ± 955.2 | 2792.5 ± 774.9 |

SD, standard deviation; n=3.

**Table S4. Substrate clearance rates dependent on CYP450 enzymes in six PHHs (μL//hr/10^6^ cells)**

| **Enzyme** | **Substrate  (Concentration µM)** | **PHH330** | **PHH409** | **PHH416** | **PHH211** | **PHH025** | **PHH789** |
| --- | --- | --- | --- | --- | --- | --- | --- |
| **CYP1A2** | Phenacetin (1.0) | 92.5 ± 23.2 | 117.5 ± 7.0 | 0.0 | 208.8 ± 14.1 | 72.4 ± 26.0 | 112.3 ± 22.4 |
| **CYP2B6** | Bupropion (1.0) | 146.8 ± 24.3 | 185.9 ± 24.1 | 200.2 ± 32.8 | 286.6 ± 17.8 | 370.6 ± 19.9 | 476.8 ± 48.2 |
| **CYP2C9** | Diclofenac (1.0) | 78.6 ± 18.9 | 162.7 ± 15.1 | 236.5 ± 17.5 | 170.7 ± 8.0 | 86.1 ± 19.9 | 246.7 ± 10.4 |
| **CYP2C19** | Mephenytoin (1.0) | 0.0 | 0.0 | 0.0 | 34.8 ± 8.6 | 48.4 ± 2.8 | 255.1 ± 64.6 |
| **CYP2D6** | Dextromethorphan (1.0) | 72.1 ± 46.7 | 273.8 ± 41.0 | 566.6 ± 21.7 | 238.4 ± 18.0 | 437.6 ± 14.4 | 902.9 ± 125.6 |
| **CYP3A4** | Testosterone (1.0) | 970.6 ± 134.4 | 1377.8 ± 58.4 | 1127.9 ± 16.4 | 1354.0 ± 67.0 | 802.7 ± 70.8 | 1981.0 ± 314.7 |

Data: mean ± standard deviation; n=3.

**Table S5. Metabolite formation rates dependent on CYP450 enzymes in six PHHs (pmol/hr/10^6^ cells)**

| **Enzyme** | **Metabolite** | **PHH330** | **PHH409** | **PHH416** | **PHH211** | **PHH025** | **PHH789** |
| --- | --- | --- | --- | --- | --- | --- | --- |
| **CYP1A2** | 4-Acetamidophenol | 74.2 ± 6.4 | 353.0 ± 14.2 | 33.9 ± 5.6 | 317.0 ± 41.8 | 0.0 | 152.2 ± 2.8 |
| **CYP2B6** | 4-Hydroxybupropion | 769.5 ± 174.4 | 370.7 ± 41.8 | 236.4 ± 11.6 | 431.4 ± 37.8 | 26.7 ± 0.6 | 485.2 ± 19.3 |
| **CYP2C9** | 4'-Hydroxydiclofenac | 102.7 ± 17.7 | 433.2 ± 43.3 | 129.9 ± 5.9 | 137.0 ± 7.4 | 0.0 | 744.7 ± 50.4 |
| **CYP2C19** | 4-Hydroxymephenytoin | 0.0 | 0.0 | 15.2 ± 0.1 | 32.7 ± 8.4 | 0.0 | 211.7 ± 16.4 |
| **CYP2D6** | Dextrorphan | 67.7 ± 6.3 | 398.2 ± 3.7 | 849.9 ± 46.9 | 478.2 ± 33.7 | 197.8 ± 20.3 | 330.0 ± 2.8 |
| **CYP3A4** | 6β-hydroxytestosterone | 2008.6 ± 147.9 | 420.5 ± 45.6 | 234.8 ± 38.3 | 479.2 ± 54.1 | 0.0 | 985.8 ± 21.3 |

Data: mean ± standard deviation; n=3.

**Table S6. Detection parameters in LC-MS for CYP450-related drug-metabolizing function and bile secretion index**


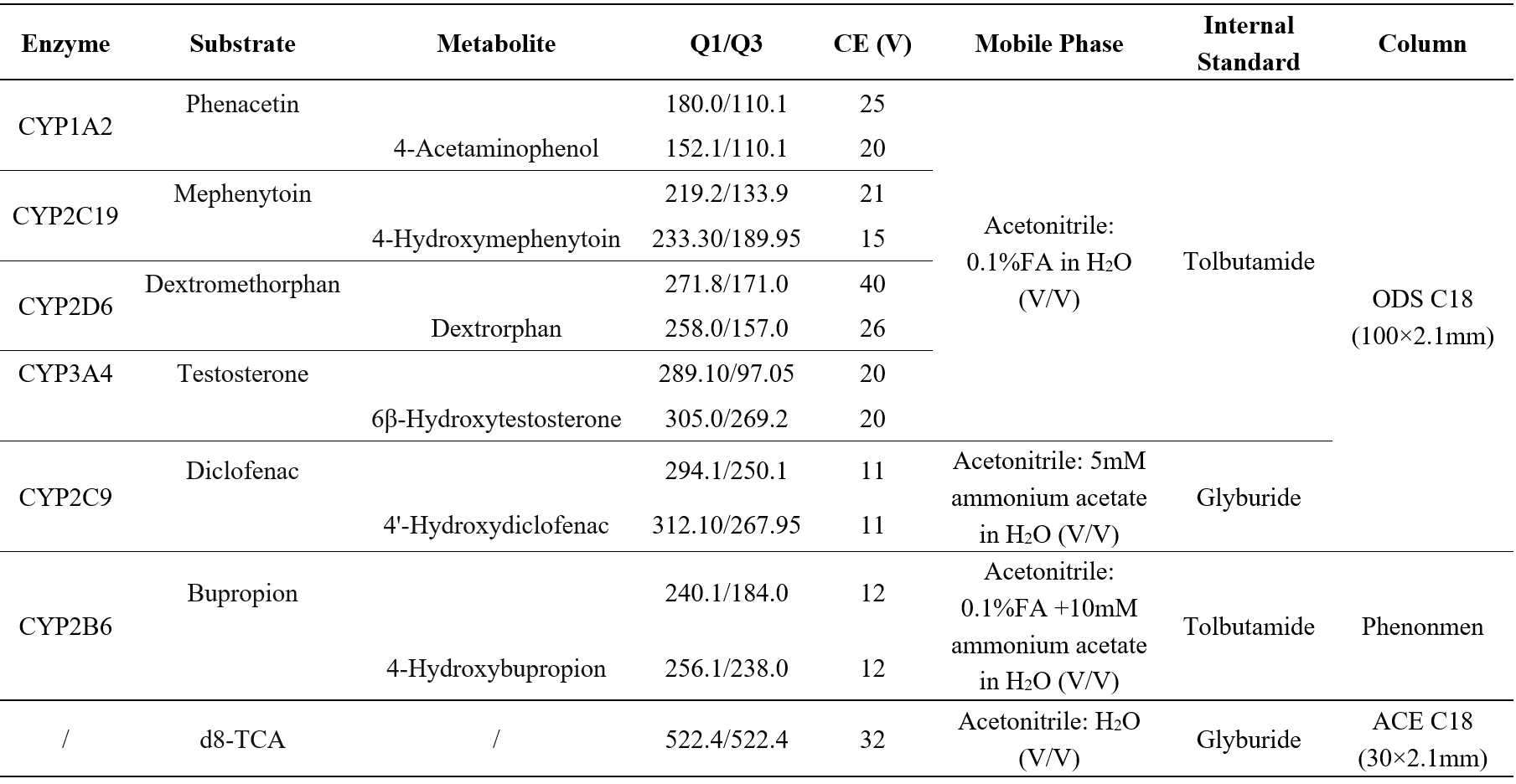


**Table S7. Bile secretion index (BEI) in PHHs by LC-MS**

|  | **PHH393** | **PHH396** | **PHH416** | **PHH005** | **PHH910** |
| --- | --- | --- | --- | --- | --- |
| **BEI (%)** | 27.2 | 27.5 | 25.7 | 62.0 | 59.0 |


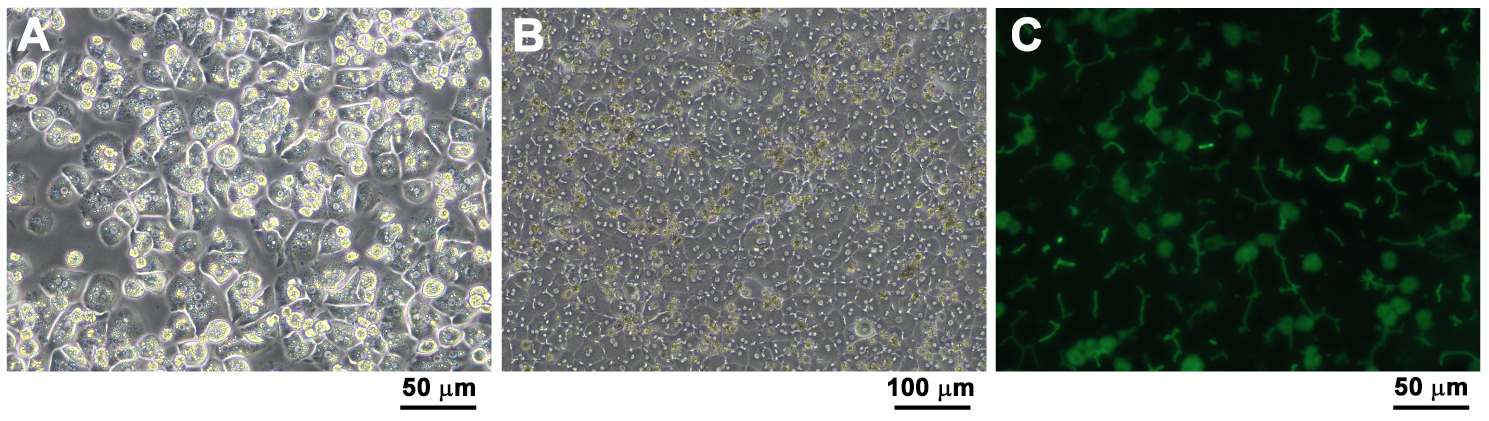


**Fig. S1** Representative graphs of PHHs (Batch: PHH005). **A** Photograph of PHHs four hours after plating in 2D-culture condition. **B** Photograph of PHHs five days after plating in sandwich-culture condition. **C** The formation of bile canaliculi networks in polarized hepatocytes were visualized by fluorescence from CDFDA (5-(and-6)-carboxy-2′, 7′-dichlorofluorescein diacetate).


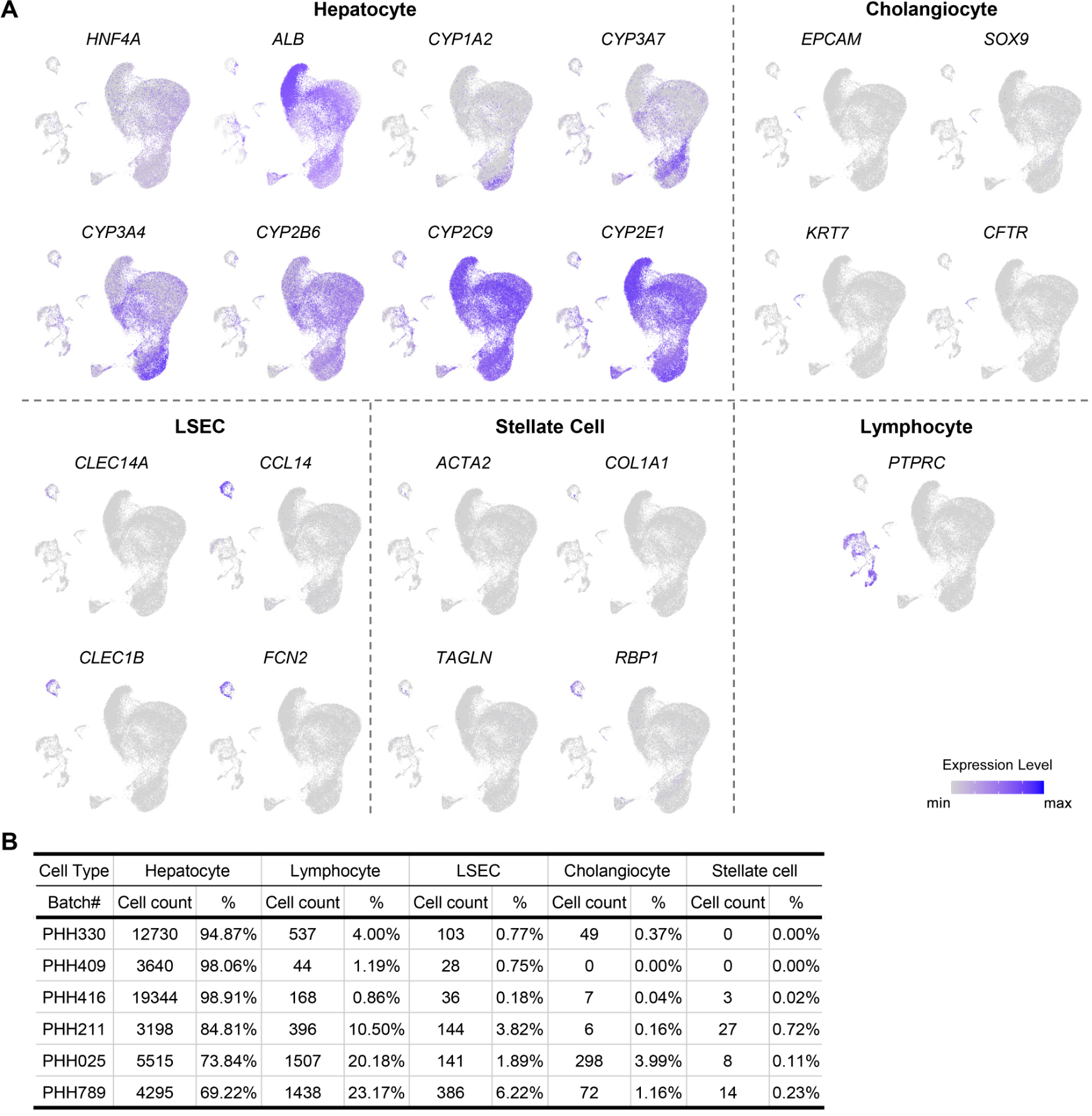


**Fig. S2** Identification and distribution of different cell types in six PHHs. **A** Dot plots showing the expression levels of indicated marker genes for different cell types in UMAP clustering. **B** Cell counts and proportions of different cell types in six PHHs.


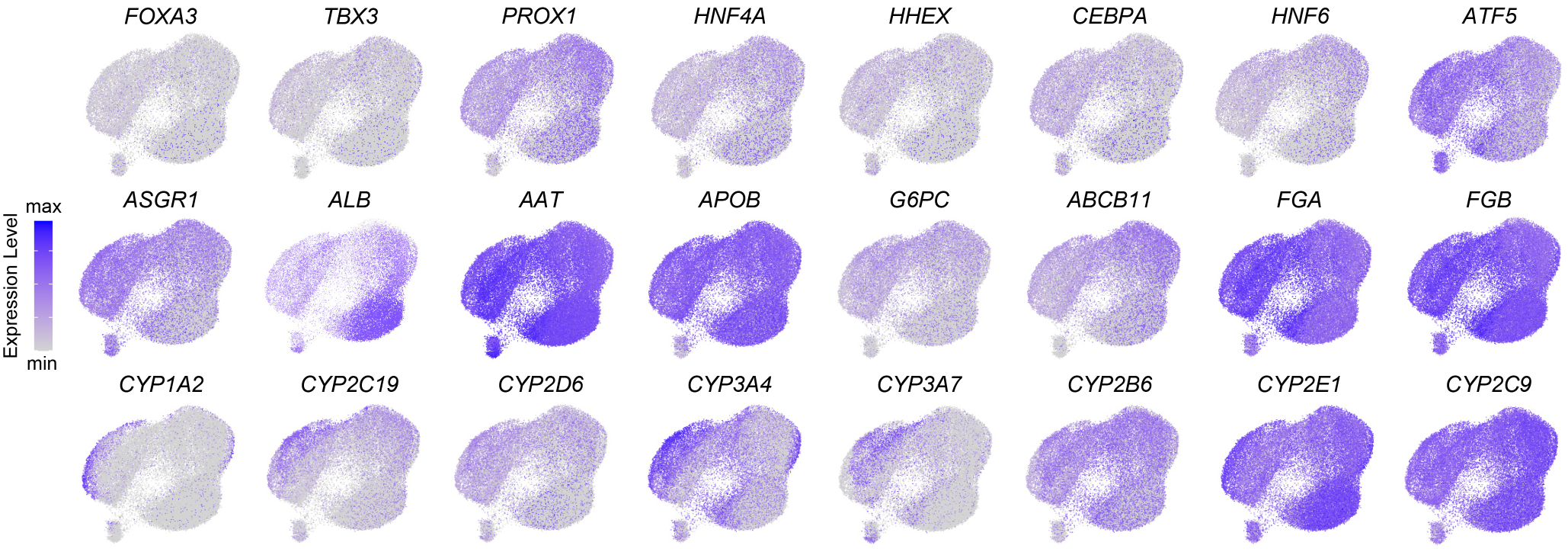


**Fig. S3** Dot plots showing the expression levels of hepatic marker genes for hepatocyte population in UMAP clustering.


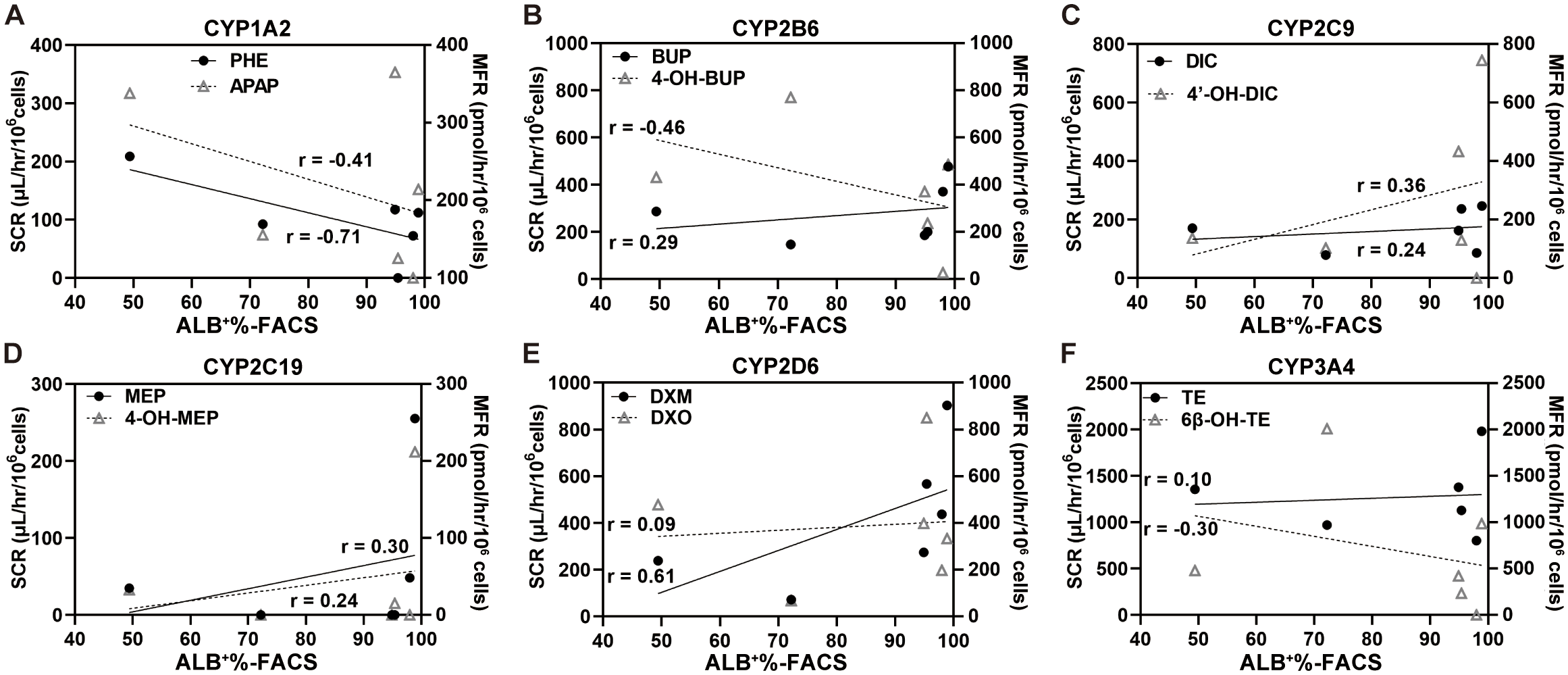


**Fig. S4** Correlations between ALB^+^% by FACS and the corresponding substrate clearance rates (SCR; solid dot or solid line) or metabolite formation rates (MFR; hollow triangle or dotted line) in six PHHs. r, Pearson correlation coefficient.


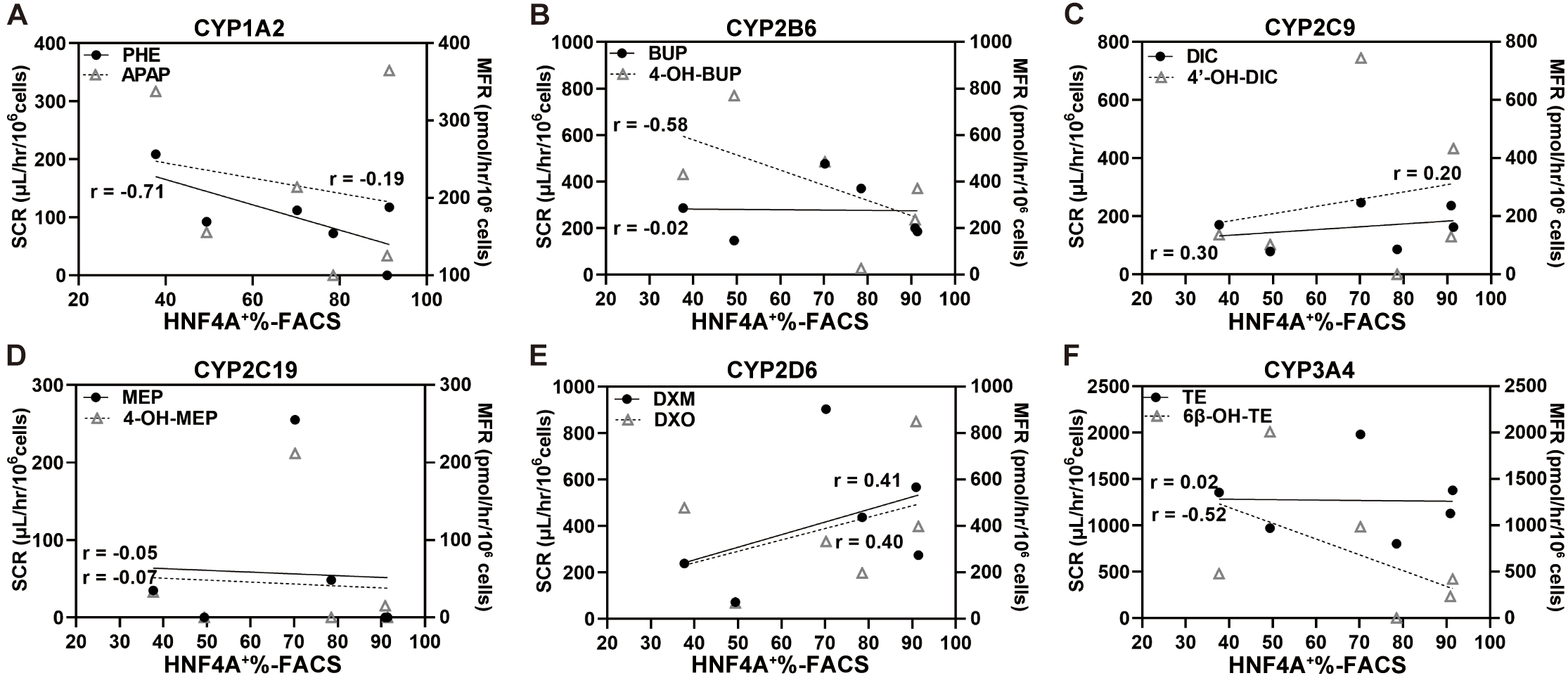


**Fig. S5** Correlations between HNF4A^+^% by FACS and the corresponding substrate clearance rates (SCR; solid dot or solid line) or metabolite formation rates (MFR; hollow triangle or dotted line) in six PHHs. r, Pearson correlation coefficient.


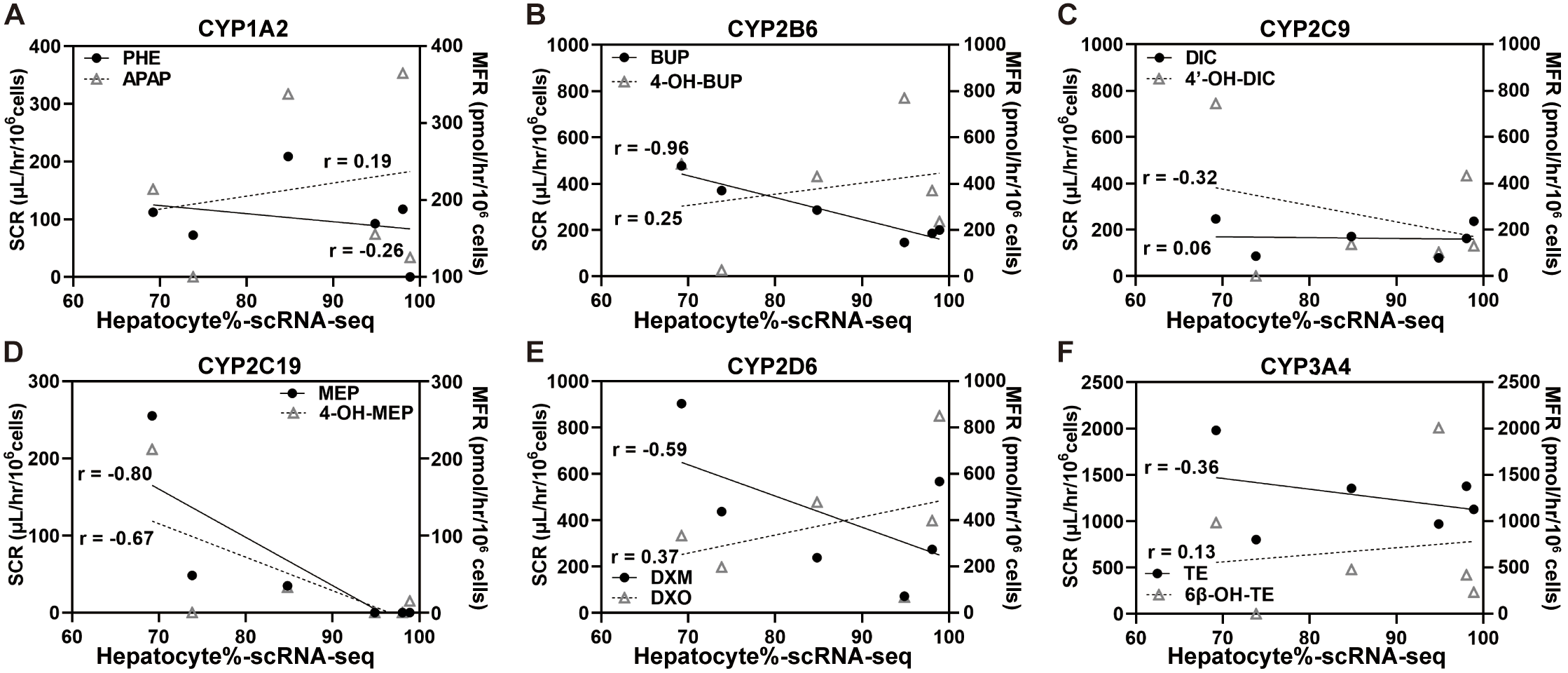


**Fig. S6** Correlations between hepatocyte% by scRNA-seq and the corresponding substrate clearance rates (SCR; solid dot or solid line) or metabolite formation rates (MFR; hollow triangle or dotted line) in six PHHs. r, Pearson correlation coefficient.


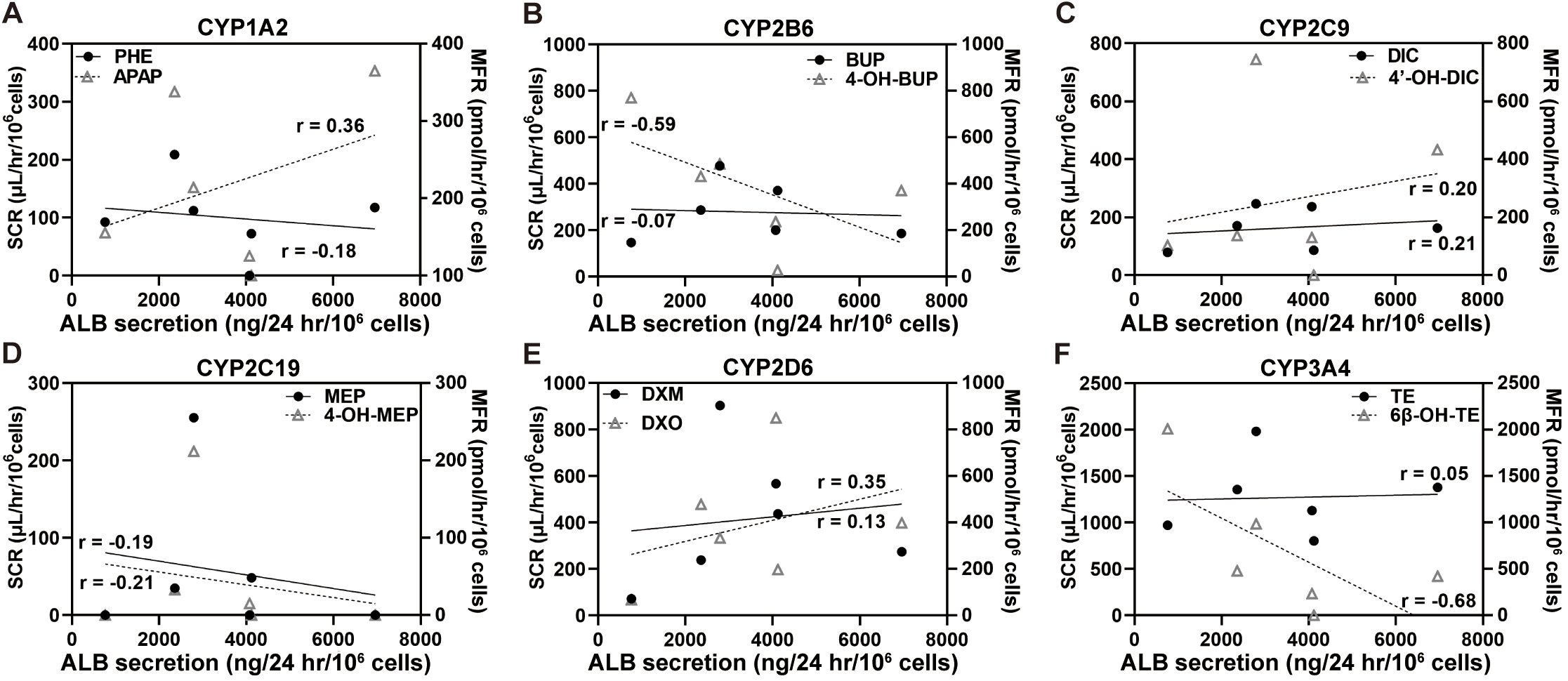


**Fig. S7** Correlations between secreted ALB level by ELISA and the corresponding substrate clearance rates (SCR; solid dot or solid line) or metabolite formation rates (MFR; hollow triangle or dotted line) in six PHHs. r, Pearson correlation coefficient.
